# One question might be capable of replacing the Shoulder Pain and Disability Index (SPADI) when measuring disability: a prospective cohort study

**DOI:** 10.1007/s11136-017-1698-y

**Published:** 2017-09-07

**Authors:** Marloes Thoomes-de Graaf, Wendy Scholten-Peeters, Yasmaine Karel, Annemieke Verwoerd, Bart Koes, Arianne Verhagen

**Affiliations:** 1 Department of General Practice, Erasmus Medical Centre, Rotterdam, The Netherlands; 2 Research group diagnostics, Avans University of Applied Science, Breda, The Netherlands; 3 Department of Human Movement Sciences, Faculty of Behaviour and Movement Sciences, Vrije Universiteit Amsterdam, MOVE Research Institute, Amsterdam, The Netherlands

**Keywords:** SPADI, Single question, Disability, Shoulder, Questionnaire

## Abstract

**Questions:**

Is it possible to replace the Shoulder Pain and Disability Index (SPADI) with a single substitute question for people with shoulder pain, when measuring disability and how well does this substitute question perform as a predictor for recovery.

**Design:**

A prospective cohort study.

**Participants:**

A total of 356 patients with shoulder pain in primary care.

**Analyses:**

Convergent, divergent, and “known” groups validity were assessed by using hypotheses testing. Responsiveness was assessed using the Receiver Operating Curve and hypothesis testing. In addition, we performed multivariate regression to assess if the substitute question showed similar properties as the SPADI and if it affected the model itself, using recovery as an outcome.

**Results:**

The Spearman correlation coefficient between the total SPADI score and the substitute question was high, and moderate with the Shoulder Disability Questionnaire. The correlation between the substitute question and the EQ-5D-3L was low and the responsiveness was acceptable. The substitute question did not significantly contribute to both prognostic prediction models as opposed to the SPADI. Regardless all models showed poor to fair discrimination.

**Conclusion:**

The single question is a reasonable substitute for the SPADI and can be used as a screening instrument for shoulder disability in primary clinical practice. It has slightly poorer predictive power and should therefore not be used for prognosis.

## Introduction

Activity limitations are one of the most important health consequences for patients with shoulder pain [1]. Activity limitations can range from difficulties with opening a jar and getting dressed, to impeding sleep [2]. Shoulder pain presents an economic burden on society due to costs of sick leave and health care and also impacts patient’s quality of life [3]. As such, health-related patient-reported outcome measures (PROMs) that assess perceived activity limitations are useful in terms of assessing the physical impairment in patients with shoulder pain [1, 4].

Both the Shoulder Pain and Disability Index (SPADI) as the Shoulder Disability Questionnaire (SDQ) are PROMs focusing on activity limitations. Several (systematic) reviews have encouraged the use of the SPADI in both clinical and research settings [5–7].

A survey among physical therapists (PTs) concluded that PROMs are most often used to ensure quality of care, to communicate with other health care providers, and to determine progress (outcomes) of individual patients [8]. These findings are consistent among other health care professionals [9]. Apart from this, a PROM can be used to predict recovery. For example, there is consistent evidence that a high level of disability is one of the predictors of poor recovery for patients with shoulder pain [10].

Nevertheless, PROMs are not (fully) integrated into clinical practice yet. A survey among nearly 500 PTs concluded that only half of them regularly used a PROM during their work [8]; this is consistent with other health care providers [11]. The most common reason for not using PROMs is that it is too time consuming for patients to complete (43%) and for clinicians to analyze, calculate, and score (30%); moreover, several PROMs are too difficult for patients to complete independently (29.1%) [8]. Even the PTs that do use PROMs during their work agreed (more than 75%) with the problems described by the non-users and also stated that PROMs are often confusing to patients.

Several initiatives have been started as a response to these concerns to facilitate the integration of PROMs in clinical care. Clinicians prefer PROMs that can be completed quickly (70%) [8]. Therefore, modifications and abbreviations of several PROMs have been developed and validated [12, 13]. Recently, the Patient-Reported Outcomes Measurement Information System (PROMIS) was developed using sample qualitative input from patients and specific analyzing methods (item response theory), to construct and evaluate a preliminary item bank to measure physical functioning [14]. Computer-adaptive testing has tremendous potential for a quick and precise PROM assessment, with significantly reduced burden for patients and clinicians [15]. Another initiative is the development of single substitute questions; recently, a study concluded that it may be feasible to replace the Tampa Scale for Kinesiophobia by a single substitute question for predicting outcome in people with sciatica in primary care [16].

We therefore aimed to develop and evaluate the validity, responsiveness, and predictive power of a single substitute question for the SPADI as this might be helpful to integrate a PROM into clinical practice.

## Methods

### Design

This is a secondary analysis of a prospective cohort study (ShoCoDiP-study), including patients with shoulder pain in physiotherapy setting. Aims of the ShoCoDiP-study were e.g., to evaluate physiotherapy care and prognostic factors in patients with shoulder pain and investigate whether Musculoskeletal ultrasound and the working alliance are related to patient recovery. Details of the design are presented elsewhere [17]. The Medical Ethics Committee of the Erasmus Medical Center in Rotterdam approved the study (MEC-2011-414). Informed consent was obtained from all patients.

### Study population

Patients were recruited from primary care physical therapy clinics between November 2011 and December 2012. Patients with shoulder pain were eligible for inclusion if they were at least 18 years old and adequately understood the Dutch language. Patients with serious pathology (infection, cancer or fracture), previous surgery or diagnostic imaging techniques of the shoulder, such as Magnetic Resonance Imaging or Ultrasound in the previous 3 months, were excluded [17].

### Development of the substitute question

In a focus meeting with the ShoCoDiP-project team (consisting of physical therapists, manual therapists, general practitioners, a radiologist, an orthopedic surgeon, and epidemiologists), various items were discussed that could act as a substitute question to cover the entire domain of the SPADI questionnaire. The final substitute question was chosen based on consensus within the research team: “Please state the amount of limitation in daily activity you experience due to your shoulder pain.” This question could be answered on an 11-point scale, where 0 = no limitation at all and 10 = completely disabled.

### Baseline measurement

Participating patients received an online questionnaire that included items focused on demographic characteristics, pain intensity [Numeric Rating Scale (NRS)], disability (the SDQ, SPADI and substitute question), and health-related quality of life (EQ-5D-3L).

#### Pain intensity

The 11-point NRS was used to capture the patient’s pain intensity. The scale is anchored from “no pain” to “worst imaginable pain.” Patients rate their current level of pain and their worst and least amount of pain in the last 24 h. The NRS has shown to be valid, reliable, and responsive in patients with shoulder pain [4].

*The SPADI* is a self-administered questionnaire designed to measure pain and disability associated with shoulder pain. It consists of 13 items and each question refers to the past week. Five items measure severity/intensity of pain, and eight items measure disability. Items can be scored on a scale ranging from 0 to 10, where 0 represents “no pain/no difficulty” and 10 “worst pain imaginable/so difficult it requires help” [18, 19]. The total score varies between 0 and 100, a higher score indicates a higher level of pain-related disability [18]. The Dutch SPADI (SPADI-D) has shown to be valid (hypothesis testing, factor structure), reliable (internal consistency and test–retest), interpretable (measurement error, floor, and ceiling effects) and responsive, in patients with shoulder pain in primary care [20, 21].

*The SDQ* is a pain-related disability questionnaire developed in Dutch, which consists of 16 items [1, 22]. All items refer to the preceding 24 h. Response options are “yes,” “no,” or “not applicable.” The option “not applicable” indicates the situation that the issue has not occurred in the past 24 h. The SDQ-score can range from 0 to 100 with a higher score indicating more severe disability [1, 22]. The SDQ is a valid and responsive measure [1, 23].

*The EQ-5D-3L* is a health-related quality of life questionnaire covering five dimensions of health: mobility, self-care, usual activities, pain/discomfort, and anxiety/depression [24]. Response options are “no problems,” “some problems,” “extreme problems.” The Dutch version is an official language version [24].

### Follow-up

All patients received the SPADI-D, the SDQ, the substitute question, and the Global Perceived Effect (GPE)-scale 26 weeks after initial presentation. Within this period, the patient received individualized physical therapy treatment for 1 or more sessions. Outcome measure was perceived recovery by the patient, measuring with the GPE-scale. The GPE-scale is a 7-point scale scoring whether the patient’s condition has improved or deteriorated. This scale ranges from “completely recovered” to “worse than ever.” The GPE-scale has good test–retest reliability and correlates well with changes in pain and disability [25].

### Analysis

All statistical analyses were performed with SPSS 23. For this study, all patients that did not answer the substitute question were excluded. Handling of missing items for the SPADI and SDQ was performed as described by the original authors [18, 23]. This means that patients were excluded from the analysis if there were more than two items missing per SPADI-subscale [18] or when more than two items were missing from the SDQ [23]. The total score of the questionnaires for the included patients were calculated by adding up the item scores and dividing them only by the number of items that were answered and deemed applicable to the subject [18, 23].

All data were checked on normality, using a Stem-and-leaf Plot, Q-Plot and Whisker box. Non-parametric tests were used if data were not normally distributed. Descriptive statistics were used to calculate frequencies.

#### Validity

##### Correlations and hypotheses

Correlations were calculated using the Pearson correlation coefficient in case of a normal distribution of the data, otherwise a Spearman correlation coefficient was used. Correlations were rated as follows: *r* < 0.30 as low (a negligible correlation); 0.30 ≤ *r* < 0.45 as moderate; 0.45 ≤ *r* < 0.60 as substantial and *r* ≥ 0.60 as high [26].

*Convergent validity* relates to the extent to which a particular instrument corresponds to the construct (theoretical concept) of shoulder pain and function [27]. As the substitute question is designed to possibly replace the SPADI, we hypothesize that the correlation between substitute question and the total score of the SPADI is high (*r* ≥ 0.60). We also measured the correlation between the substitute question and the SDQ, as the instruments are based on a similar construct, we expected a high correlation as well, but lower than the correlation with the SPADI (as the substitute question is designed to replace the SPADI). The SDQ has a different type of answering option and the focus of the SDQ lies on “pain during an activity,” as opposed to the SPADI of which the majority of questions is focussed on “difficulties with performing an activity due to pain.” We therefore expected the substitute question to be highly correlated (*r* > 0.60) with the SPADI and substantially correlated (*r* between 0.45 and 0.60) with the SDQ [27].

*Divergent validity* relates to the extent to which a particular instrument does not correspond to the construct (theoretical concept) of shoulder pain and function. As two items of the EQ-5D-3L and the substitute question are based on different constructs (the mobility-item and the item anxiety/depression), we expect the correlation coefficient between both to be low (*r* < 0.30) [27].

*Known groups validity* We assumed that patients with high initial pain (>7 on the Numeric Rating Scale in the preceding 24 h) and work absence would have a higher level of perceived disability. Both groups had been chosen a priory. The independent sample Mann–Whitney *U* test was used to test the difference between known groups.

#### Responsiveness

Responsiveness was assessed using the area under the ROC curve (AUC) and hypothesis testing. Patients were selected if they completed the SPADI-D and the substitute question at baseline and follow-up and the GPE-scale at follow-up at 26 weeks.

##### AUC method

We calculated the AUC to assess the ability of the substitute question to discriminate between patients who are considered improved and not importantly changed according to the GPE, using a frequently used anchor and considered patients as recovered when they answered they were ‘completely recovered’ or ‘much improved’ and as not importantly improved when they answered ‘slightly improved,’ ‘no change,’ or ‘slightly worse’ [28–30].

A benchmark that has been previously used to establish that outcome measures are useful in discriminating improved and unimproved patients has been set at 0.70 AUC [31].

##### Hypothesis testing

Hypothesis testing for responsiveness was based on the concept that the correlation between the change score of related constructs (SPADI) must be high. Hypothesis testing was quantified by the Pearson correlation coefficient in case of a normal distribution of the data and otherwise a Spearman correlation coefficient was used. Correlation coefficients between the substitute change score and the SPADI change score were expected to be above 0.50 [32]. A substantial correlation (*r* between 0.45 and 0.60) was also expected between the change score of substitute question and the change score of the SPDQ and the GPE-scale. Correlations between the change score of the substitute question and the change score of EQ-5D-3L mobility as well as the anxiety/depression item were expected to be low (*r* < 0.30).

#### Predictive power

Multivariate logistic regression analysis was used to predict recovery after 26 weeks. All assumptions (linearity between independent variables and log odds and multicollinearity (>0.80) for continuous variables) were checked before model building. We included no more than one independent variable per ten events (for the smallest outcome group) in the multivariable analysis [33].

##### Basic model

A systematic review concluded that there was moderate to strong evidence that high pain intensity, increasing age, a longer duration of complaints, and high disability at baseline predict a poorer outcome in patients with shoulder pain [10]. Another review concluded that higher age, a longer duration of shoulder pain, and high disability were associated with poor recovery [34].

Patients were selected if they completed the GPE-scale at follow-up at 26 weeks and all items of interest at baseline (age, duration of complaints, pain intensity, the substitute question, and the SPADI). We checked if there were significant differences in the relevant characteristics between the patients selected in this analysis and those excluded.

Initially, three different models were built. The first model included all predictors (age, duration of complaints, and pain intensity) retrieved from the systematic reviews [10, 34]. In the second model, we added the SPADI and in model 3 we added the substitute question to model 1.

##### Sensitivity analysis

A sensitivity analysis (model 4) was performed by adding relevant prognostic factors as found in our own analysis in the total cohort [35] and not in systematic reviews (no depression or anxiety, a paid job and good working alliance [measured with the working alliance inventory (WAI)]. We chose to exclude the WAI, as the total score of the WAI was only available for 64 patients. We added the SPADI to the basic sensitivity model in model 5 and added the substitute question in model 6.

We assessed the prognostic power (Nagelkerke *R* ^2^), the discriminative ability (AUC), and the reliability of the models (Hosmer and Lemeshow). We considered a comparable (<15% difference) overall correct percentage and Nagelkerke *R* ^2^ in model 2 and 3, as an indication that it might be valid to replace the questionnaire by its substitute question in predicting outcome. An AUC can be categorized into four categories: poor discrimination (between 0.5 and 0.7), fair discrimination (between 0.7 and 0.8), acceptable discrimination (AUC > 0.8), whereas an AUC of 1.0 indicates perfect discrimination [36]. Hosmer and Lemeshow goodness of fit tests were used to assess whether or not the observed event rates match the expected event rates in subgroups of the model population, a good model fit is indicated by a non-significant result. The −2loglikelihood is the equivalent of the residuals; a lower value is a better fit.

Furthermore, we checked whether or not the total score from the SPADI and the substitute question contributed significantly to the original model (model 1), using the *χ* ^2^ test.

We repeated this process for the sensitivity analysis with different predictors (model 4–6).

## Results

### Patient characteristics

A total of 389 patients responded in our cohort study, 19 of them did not return the SPADI at baseline. We excluded another 14 patients due to too many missing data on the SPADI or SDQ. Of these 356 patients, all answered the substitute question and were therefore included in this study. Demographic characteristics are presented in Table 1, the mean age of the patients was 49.5 (SD 13) years and 47% was male. Of these 356 patients, 250 completed the GPE after 26 weeks and answered all items of interest at baseline (age, duration of complaints, NRS and the SPADI according to the missing item criteria and the substitute question). Responsiveness was based on 237 patients answering the substitute question at baseline and follow-up and the GPE-scale.

**Table 1:** Baseline characteristics

Population	Total cohort (*n* = 356)	Cohort “Follow-up” (*n* = 250)	Not included in the predictive study (*n* = 106)	*p* value
Gender (male) (%)	166 (47%)	116 (46%)	50 (47%)	0.894
Age Mean (SD)	49.5 (13.1)	50.2 (13.0)	47.8 (13.1)	0.118
SPADI score (0–100) Mean (SD)	46.7 (21.3)	47.5 (21.2)	45.0 (21.7)	0.310
Substitute question (0–10) Median (IQR)	4 (2–6)	4 (2–6)	3.5 (1–6)	0.549
Duration of complaints in weeks Median (IQR)	12 (6–26)	12 (6–26)	12 (6–24)	0.502
Use of medication (%)	171 (49%)	129 (52%)	42 (40%)	0.055
Pain intensity (NRS) (0–10) Median (IQR)	6 (4–7)	6 (4–7)	5 (4–7)	0.068

The data of the substitute question were not normally distributed. The median score of the substitute question was 4 points with an interquartile range (IQR) from 2 to 6. The SPADI was normally distributed and had a mean of 46.7 (21.3).

As it is unusual to compare data presented in different ways, we also presented the median of the SPADI (median 48.7, IQR 28.8–65.0) in order to facilitate a swift visual inspection of the score of the question of interest (the substitute question) and the score of the total SPADI.

### Validity

#### Convergent validity

The Spearman correlation coefficient between the substitute question and the total SPADI score was 0.74 and with the SDQ 0.59. Our hypotheses were confirmed as the substitute question showed a high correlation with the SPADI and a substantial correlation with the SDQ.

#### Divergent validity

The spearman correlation between the substitute question and the mobility-item of the EQ-5D-3L was 0.23 and with the item anxiety/depression 0.20. Our hypotheses were hereby confirmed as the correlation was low between the instruments that measure a different construct and the substitute question.

#### Known groups validity

Differences between “known groups” were statistically significant (Table 2).

**Table 2:** Known groups validity

Group	Median score substitute question	*p* value
Pain (*n* = 356)		
High initial pain > 7	6 (4–7)	0.000
Low initial pain < 7	3 (1–5)	
Work absence (*n* = 318)		
Work absence due to shoulder pain	6 (5–7)	0.000
No work absence due to shoulder pain	3 (1–5.25)	

### Responsiveness

**Fig. 1:**
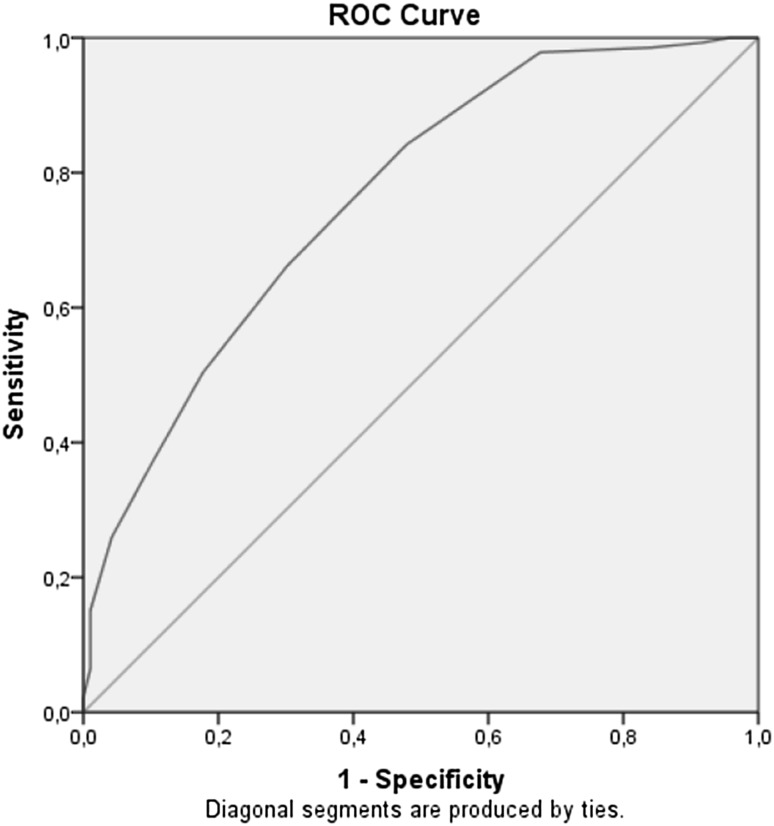
ROC curve based upon the GPE

The AUC was 0.76 with a 95% confidence interval ranging from 0.70 to 0.83. Figure 1 shows the ROC curve based upon the GPE.

Hypothesis testing for responsiveness resulted in a Spearman correlation between the SPADI-D change score and the substitute change score of 0.71 and 0.60 with the SDQ change score. The spearman correlation between the GPE and the substitute question was 0.47. The Spearman correlation between the substitute question and both the mobility as the anxiety/depression item of the EQ-5D-3L was 0.10.

Based on the AUC values and confirmation of the hypothesis, we consider the substitute question to be a responsive measurement instrument.

### Prediction model

There were no significant differences in the relevant characteristics between the patients selected in this analysis (*n* = 250) and those excluded (*n* = 106) (Table 1).

Out of 250 patients, 150 patients were labeled as recovered after 26 weeks. For all variables included in the model, the variance inflation factors were <1.5 and correlation coefficients <0.8, suggesting that no linearity and multicollinearity was present.

Table 3 shows the predictive models. Model 1 consisted of the following variables: age, pain, and duration of complaints. The correct overall percentage was 64.8% and the Nagelkerke *R* ^2^ was 0.90.

**Table 3:** Predictive value

Predictors for recovery	Model 1 (*n* = 250)	Model 2 (*n* = 250)	Model 3 (*n* = 250)
	OR (95% CI)	OR (95% CI)	OR (95% CI)
Age (younger)	0.98 (0.96–1.00)	0.98 (0.96–1.01)	0.98 (0.96–1.00)
Duration of complaints (in weeks) (shorter)	0.99 (0.99–1.00)	0.99 (0.99–1.00)	0.99 (0.99–1.00)
Pain using an NRS (lower levels of pain)	0.92 (0.80–1.05)	1.02 (0.87–1.21)	0.97 (0.83–1.13)
Disability using the total SPADI score (lower level of functional disability)		0.98 (0.97–1.00)	
Disability using the substitute question (lower level of functional disability)			0.92 (0.81–1.04)
Performance of the model			
Correct overall percentage	64.8%	65.6%	65.2%
Nagelkerke *R* ^2^	0.090	0.114	0.098
AUC (95% CI)	0.64 (0.57–0.72)	0.66 (0.59–0.73)	0.65 (0.58–0.72)
Hosmer and Lemeshow	0.757	0.875	0.553
−2 Log likelihood	319.286	314.534	317.594

Model 1 age, duration of complaints and pain; Model 2 age, duration of complaints, pain and the SPADI; Model 3 age, duration of complaints, pain and the substitute question

Model 2 consisted of the following variables: age, pain, duration of complaints, and the SPADI. The Chi-Square test for adding the SPADI was significant (*p* = 0.029).

Model 3 consisted of the following variables: age, pain, duration of complaints, and the substitute question. The *χ* ^2^ test for adding the substitute question was not significant (*p* = 0.193).

All three models showed poor discrimination and the AUC values were within the 95% CI intervals of each other. Differences between both models were small (Table 3). The largest differences were found between the Hosmer and Lemeshow goodness of fit of model 2 and 3; however, both were non-significant. The odds of the SPADI and the substitute question were quite exchangeable; however, the confidence interval of the substitute question was wider.

#### Sensitivity analysis

The basic model (model 4) consisting of age, duration of complaints, pain, employment and not being depressed and was based on 241 patients, as nine patients had a missing value regarding employment or depression. The correct overall percentage was 63.9% and the Nagelkerke *R* ^2^ was 0.127.

Model 5 included all predictors plus the SPADI. The *χ* ^2^ Omnibus test for adding the SPADI was significant (*p* = 0.039).

Model 6 included all predictors plus the substitute question. The *χ* ^2^ test for adding the substitute question was not significant (*p* = 0.501) Table 4.

**Table 4:** Sensitivity analysis

Predictors for recovery	Model 4 (*n* = 241)	Model 5 (*n* = 241)	Model 6 (*n* = 241)
	OR (95% CI)	OR (95% CI) *p* value	OR (95% CI) *p* value
Having a job	1.77 (0.87–3.62)	1.80 (0.88–3.68)	1.75 (0.85–3.57)
Being depressed (not being depressed helps to recover)	0.41 (0.20–0.85)	0.42 (0.21–0.88)	0.43 (0.21–0.89)
Age (younger)	0.99 (0.97–1.02)	0.99 (0.96–1.02)	0.99 (0.97–1.02)
Duration of complaints (in weeks) (shorter)	0.99 (0.99–1.00)	0.99 (0.99–1.00)	0.99 (0.99–1.00)
Pain using an NRS (lower levels of pain)	0.95 (0.83–1.09)	1.06 (0.89–1.27)	0.98 (0.83–1.14)
Lower disability (SPADI total score)		0.98 (0.97–1.00)	
Lower disability (substitute question)			0.96 (0.84–1.09)
Performance of the model			
Correct overall percentage	63.9%	66.0%	66.8%
Nagelkerke *R* ^2^	0.127	0.149	0.130
AUC (95% CI)	0.67 (0.60–0.74)	0.69 (0.62–0.75)	0.68 (0.61–0.74)
Hosmer and Lemeshow	0.310	0.853	0.051
−2 Log likelihood	301.001	296.753	300.547

Model 4 age, duration of complaints, pain, depression and being employed; Model 5 age, duration of complaints, pain, depression, being employed, the SPADI; Model 6 age, duration of complaints, pain, depression, being employed, the substitute question

All models showed poor discrimination, with small differences. The largest differences were found between the Hosmer and Lemeshow goodness of fit of model 4 and 5; however, both were non-significant. The odds of the SPADI and the substitute question were again quite exchangeable; however, the confidence interval of the substitute question was wider.

## Discussion

Measurement with the single question can be completed in a shorter amount of time as compared with the SPADI, which takes about 3 min to complete. This could have impact on the use of the instrument in clinical practice and increase the integration of patient-reported outcome measures (PROMs), as the most common reasons for not using them are that they are too time consuming for patients to complete and too time consuming for clinicians to analyze. Quality of life research revealed that both single questions and multi-item scales have a high potential as well as some disadvantages at the same time [37]. They stated that the two types of indices are not mutually exclusive and can be used together in a single research study or in a clinical setting. Single items have the advantage of simplicity at the cost of detail [37]. Multiple-item indices have the advantage of providing a complete profile of quality of life component constructs at the cost of increased burden and of asking potentially irrelevant questions [37].

However, the predictive power of the substitute question is not entirely equal to the SPADI as the substitute question did not significantly contribute to both models according to the Chi-Square test, as opposed to the SPADI. Regardless, switching between the SPADI and the substitute question did not have a great impact on the AUC, as all models (with the SPADI and the substitute question) showed poor discrimination. The predictive power of the model including the substitute question for predicting recovery was slightly lower (10%) compared to the model with the SPADI (13%), which are both poor. As these prediction models should be used carefully, this especially applies to using the substitute question as a predictor.

### Comparison to the literature

Not many studies have been published regarding a substitute question. One study reported that a single self-reported question to assess habitual physical activity is valid and responsive to change and thus useful for epidemiological research in community-dwelling older people, also in follow-up studies. They found correlations between self-reported habitual physical activity and mobility and accelerometer-based physical activity variables [38]. Another study assessed the reliability, the specificity, and sensitivity of a single question (with a dichotomized answering option) regarding hearing impairment in elder people. The reliability of the single question was lower than the reliability of the complete questionnaire. Their conclusion was that the entire instrument was more effective in assessing the impact of a hearing impairment on quality of life than the single question [39]. A third study assessed if the use of single items of a depression questionnaire were a reasonable alternative to the total scale in chiropractic patients with low back pain. They analyzed the association between the single candidate items and outcome, as well as the predictive capacity of both the total questionnaire as the single items. The conclusion of the authors was that a single item (no. 1 or 3) was a reasonable substitute for the entire scale when screening for depression as a prognostic factor [40]. The first study that assessed validity, responsiveness, and predictive power of a substitute question compared to a complete questionnaire found a similar result with regard to the Tampa Scale for Kinesiophobia [16]. The conclusion of this manuscript was that the unique single substitute question might be able to replace the Tampa Scale.

### Strengths and limitations

This is a new type of research, which is focused on a very pragmatic solution regarding the disuse of PROMs. The population consisted of patients from primary care, a population that is very important within the health care system and where pain-related disability is a relevant issue. We had a relatively high number of included patients, although this could have been higher if we had chosen to use imputation techniques instead of excluding patients due to the missing item criteria. We chose to respect these criteria, as our aim was to assess whether or not the substitute question might be feasible to replace the SPADI, and the criteria of the PROMs itself are therefore more important than to use imputation techniques, in order to make a more steady prediction model due to the higher number of included patients. As the demographic characteristics of the included and excluded patients did not differ, it seems unlikely that there is selection bias regarding the inclusion of patients in the responsiveness and predictive power analyses. There were no remarkable deviations with regard to the patient characteristics of the complete study population compared to the target population (patients with shoulder pain in primary care) as far as we could discern, e.g., the number of participating females was higher than the number of participating males, which is in line with the gender-specific incidence [41], as was the average age [42].

Patients were asked to answer if their shoulder pain had changed since the beginning of treatment. The time between baseline and follow-up was 26 weeks, which might have influenced their recollection of their shoulder problem at the beginning. Although this is common practice, this could have an impact on the results.

Although the SPADI is designed as if it consists of two parts (pain and disability), we chose to only formulate one substitute question and to assess the correlation with the total SPADI. The theoretical deviation into two separate parts has not been confirmed in our earlier study [20]. As the majority of the SPADI questions focuses on difficulties with performing an activity due to pain, we formulated the substitute question with a similar focus (difficulties with performing an activity due to shoulder pain).

### Future research

It is important to test the content validity of the substitute question, with patients, clinicians, and experts together. Besides, the reliability, validity, responsiveness, and predictive value should be further assessed before this question can be used in clinical practice.

## Conclusion

The correlation between the substitute question and the full SPADI was relatively high. Combined with acceptable responsiveness, the substitute question can potentially be used as a screening instrument for shoulder disability in primary clinical practice. The single question has slightly poorer predictive power than the complete SPADI, and should therefore not be used for prognosis at this moment.

## References

[1] Van Der Windt DAWM, Van Der Heijden GJMG, De Winter AF, Koes BW, Deville W, Bouter LM (1998). The responsiveness of the shoulder disability questionnaire. Annals of the Rheumatic Diseases.

[2] Feleus A, Bierma-Zeinstra SM, Miedema HS, Verhaar JA, Koes BW (2008). Management in non-traumatic arm, neck and shoulder complaints: differences between diagnostic groups. European Spine Journal.

[3] Huisstede BM, Bierma-Zeinstra SM, Koes BW, Verhaar JA (2006). Incidence and prevalence of upper-extremity musculoskeletal disorders. A systematic appraisal of the literature. BMC Musculoskeletal Disorders.

[4] Mintken PE, Glynn P, Cleland JA (2009). Psychometric properties of the shortened disabilities of the Arm, Shoulder, and Hand Questionnaire (QuickDASH) and Numeric Pain Rating Scale in patients with shoulder pain. Journal of Shoulder and Elbow Surgery.

[5] Bot SD, Terwee CB, van der Windt DA, Bouter LM, Dekker J, de Vet HC (2004). Clinimetric evaluation of shoulder disability questionnaires: A systematic review of the literature. Annals of the Rheumatic Diseases.

[6] Roy JS, MacDermid JC, Woodhouse LJ (2009). Measuring shoulder function: A systematic review of four questionnaires. Arthritis and Rheumatism.

[7] Breckenridge JD, McAuley JH (2011). Shoulder Pain and Disability Index (SPADI). Journal of Physiotherapy.

[8] Jette DU, Halbert J, Iverson C, Miceli E, Shah P (2009). Use of standardized outcome measures in physical therapist practice: Perceptions and applications. Physical Therapy.

[9] Snyder CF, Aaronson NK, Choucair AK, Elliott TE, Greenhalgh J, Halyard MY, Hess R, Miller DM, Reeve BB, Santana M (2012). Implementing patient-reported outcomes assessment in clinical practice: A review of the options and considerations. Quality of Life Research.

[10] Kuijpers T, van der Windt DA, van der Heijden GJ, Bouter LM (2004). Systematic review of prognostic cohort studies on shoulder disorders. Pain.

[11] Russak SM, Croft JD, Furst DE, Hohlbauch A, Liang MH, Moreland L, Ofman JJ, Paulus H, Simon LS, Weisman M, Tugwell P, Evidence-Based Medicine Working Groups in, R (2003). The use of rheumatoid arthritis health-related quality of life patient questionnaires in clinical practice: lessons learned. Arthritis Rheum.

[12] Stratford PW, Binkley JM (1997). Measurement properties of the RM-18. A modified version of the Roland-Morris Disability Scale. Spine.

[13] Beaton DE, Wright JG, Katz JN, Upper Extremity Collaborative (2005). Development of the QuickDASH: Comparison of three item-reduction approaches. Journal of Bone and Joint Surgery.

[14] Rose M, Bjorner JB, Becker J, Fries JF, Ware JE (2008). Evaluation of a preliminary physical function item bank supported the expected advantages of the Patient-Reported Outcomes Measurement Information System (PROMIS). Journal of Clinical Epidemiology.

[15] Turner RR, Quittner AL, Parasuraman BM, Kallich JD, Cleeland CS, Mayo FDAP-ROCMG (2007). Patient-reported outcomes: Instrument development and selection issues. Value Health.

[16] Verwoerd AJ, Luijsterburg PA, Timman R, Koes BW, Verhagen AP (2012). A single question was as predictive of outcome as the Tampa Scale for Kinesiophobia in people with sciatica: An observational study. Journal of Physiotherapy.

[17] Karel YH, Scholten-Peeters WG, Thoomes-de Graaf M, Duijn E, Ottenheijm RP, Koes BW, Verhagen AP (2013). Current management and prognostic factors in physiotherapy practice for patients with shoulder pain: Design of a prospective cohort study. BMC Musculoskeletal Disorders.

[18] Roach KE, Budiman-Mak E, Songsiridej N, Lertratanakul Y (1991). Development of a shoulder pain and disability index. Arthritis Care and Research.

[19] Paul A, Lewis M, Shadforth MF, Croft PR, Van Der Windt DA, Hay EM (2004). A comparison of four shoulder-specific questionnaires in primary care. Annals of the Rheumatic Diseases.

[20] Thoomes-de Graaf M, Scholten-Peeters GG, Duijn E, Karel Y, Koes BW, Verhagen AP (2014). The Dutch Shoulder Pain and Disability Index (SPADI): A reliability and validation study. Quality of Life Research.

[21] Thoomes-de Graaf M, Scholten-Peeters W, Duijn E, Karel Y, de Vet HC, Koes B, Verhagen A (2017). The responsiveness and interpretability of the shoulder pain and disability index. Journal of Orthopaedic and Sports Physical Therapy.

[22] Jamnik H, Spevak MK (2008). Shoulder pain and disability Index: Validation of slovene version. International Journal of Rehabilitation Research.

[23] de Winter AF, van der Heijden GJMG, Scholten RJPM, van der Windt DAWM, Bouter LM (2007). The Shoulder Disability Questionnaire differentiated well between high and low disability levels in patients in primary care, in a cross-sectional study. Journal of Clinical Epidemiology.

[24] Lamers LM, McDonnell J, Stalmeier PF, Krabbe PF, Busschbach JJ (2006). The Dutch tariff: results and arguments for an effective design for national EQ-5D valuation studies. Health Economics.

[25] Kamper SJ, Ostelo RW, Knol DL, Maher CG, de Vet HC, Hancock MJ (2010). Global Perceived Effect scales provided reliable assessments of health transition in people with musculoskeletal disorders, but ratings are strongly influenced by current status. Journal of Clinical Epidemiology.

[26] Burnand B, Kernan WN, Feinstein AR (1990). Indexes and boundaries for “quantitative significance” in statistical decisions. Journal of Clinical Epidemiology.

[27] Mokkink LB, Terwee CB, Knol DL, Stratford PW, Alonso J, Patrick DL, Bouter LM, de Vet HC (2010). The COSMIN checklist for evaluating the methodological quality of studies on measurement properties: a clarification of its content. BMC Medical Research Methodology.

[28] Weenink JW, Braspenning J, Wensing M (2014). Patient reported outcome measures (PROMs) in primary care: An observational pilot study of seven generic instruments. BMC Family Practice.

[29] Luijsterburg PA, Verhagen AP, Ostelo RW, van den Hoogen HJ, Peul WC, Avezaat CJ, Koes BW (2008). Physical therapy plus general practitioners’ care versus general practitioners’ care alone for sciatica: A randomised clinical trial with a 12-month follow-up. European Spine Journal.

[30] Farrar JT, Young JP, LaMoreaux L, Werth JL, Poole RM (2001). Clinical importance of changes in chronic pain intensity measured on an 11-point numerical pain rating scale. Pain.

[31] Terwee CB, Bot SD, de Boer MR, van der Windt DA, Knol DL, Dekker J, Bouter LM, de Vet HC (2007). Quality criteria were proposed for measurement properties of health status questionnaires. Journal of Clinical Epidemiology.

[32] de Vet HC, Mokkink LB, Knol DL (2011). Practical guides to biostatistics and epidemiology.

[33] Harrell FE, Lee KL, Mark DB (1996). Multivariable prognostic models: issues in developing models, evaluating assumptions and adequacy, and measuring and reducing errors. Statistics in Medicine.

[34] Chester R, Shepstone L, Daniell H, Sweeting D, Lewis J, Jerosch-Herold C (2013). Predicting response to physiotherapy treatment for musculoskeletal shoulder pain: a systematic review. BMC Musculoskeletal Disorder.

[35] Karel YH, Verhagen AP, Thoomes-de Graaf M, Duijn E, van den Borne MP, Beumer A, Ottenheijm RP, Dinant GJ, Koes BW, Scholten-Peeters GG (2016). Development of a prognostic model for patients with shoulder complaints in physiotherapy. Physical Therapy.

[36] Hosmer DWJ, Lemeshow S, Sturdivant RX (2013). Applied logistic regression.

[37] Sloan JA, Aaronson N, Cappelleri JC, Fairclough DL, Varricchio C, Clinical Significance Consensus Meeting (2002). Assessing the clinical significance of single items relative to summated scores. Mayo Clinic Proceedings.

[38] Portegijs E, Sipila S, Viljanen A, Rantakokko M, Rantanen T (2016). Validity of a single question to assess habitual physical activity of community-dwelling older people. Scandinavian Journal of Medicine & Science in Sports.

[39] Tomioka K, Ikeda H, Hanaie K, Morikawa M, Iwamoto J, Okamoto N, Saeki K, Kurumatani N (2013). The Hearing Handicap Inventory for Elderly-Screening (HHIE-S) versus a single question: reliability, validity, and relations with quality of life measures in the elderly community, Japan. Quality of Life Research.

[40] Kongsted A, Aambakk B, Bossen S, Hestbaek L (2014). Brief screening questions for depression in chiropractic patients with low back pain: Identification of potentially useful questions and test of their predictive capacity. Chiropractic & Manual Therapies.

[41] Picavet HS, Schouten JS (2003). Musculoskeletal pain in the Netherlands: Prevalences, consequences and risk groups, the DMC(3)-study. Pain.

[42] Kooijman, M., Barten, J., Swinkels, I., & Veenhof, C. Jaarcijfers 2010 en trendcijfers 2006-2010 fysiotherapie. Landelijke Informatievoorziening Paramedische Zorg. Utrecht: NIVEL http://www.nivel.nl/lipz.

